# A Phantom Investigation of the Robustness and Adaptability of a Novel PET LINAC During Lung Cancer Biology-Guided Radiotherapy (BgRT) Characterized by Unplanned Perturbations in the Tumor Trajectory

**DOI:** 10.3390/cancers18111763

**Published:** 2026-05-28

**Authors:** Roland Teboh, Offormata Osunkwor, Brett Lewis

**Affiliations:** John Theurer Cancer Center, Hackensack University Medical Center, Hackensack, NJ 07601, USA; offormata.osunkwor@hmhn.org (O.O.); brett.lewis@hmhn.org (B.L.)

**Keywords:** lung cancer radiotherapy, F18-FDG, biology-guided radiotherapy, PET LINAC, SCINTIX therapy

## Abstract

This novel biology-guided radiotherapy system (BgRT), the RefleXion X1 SCINTIX™ system, uses annihilation photons produced from a PET-avid tumor to plan and then subsequently guide the delivery of beamlets of radiation to the tumor, tracking the dose delivered in real time. The current BgRT system is cleared by the U.S. Food and Drug Administration (FDA) to treat tumors in the lung or bone characterized by periodic or non-periodic motion, respectively. In this work, we assessed the robustness and adaptability of the dose tracking capability under tumor motion perturbation. We designed a phantom study based on a lung case planned under periodic tracking for four-pass (4-Pass) BgRT and then introduced transient translational shifts to the phantom motion continuously under treatment. By comparing the dose delivered under a normal periodic sinusoidal tumor motion to the dose delivered under a perturbed sinusoidal delivery, with a simple perturbation situation characterized by a perturbation introduced at the end of the second pass of the 4-Pass delivery and a more complex situation with perturbation introduced at each of the four passes of the 4-Pass delivery, we assessed the robustness and adaptability of the RefleXion X1 SCINTIX™ system in delivering the correct dose under such circumstances.

## Simple Summary

This study is an independent assessment of the robustness and adaptability of a novel PET LINAC to situations that would be otherwise unanticipated or specifically unaccounted for during treatment planning. The focus was on lung cancer biology-guided radiotherapy (BgRT) where perturbations in tumor trajectory are observed that were absent during planning sessions. By designing and delivering phantom experiments that incorporated the known perturbations in the BgRT workflow from simulation to treatment delivery and by measuring and comparing the doses delivered with and without the perturbations, we showed that within the limits of the perturbations studied in this work, the system would safely and accurately deliver a radiation dose to the target. More studies are needed to draw a general conclusion on the system’s robustness and adaptability to more sophisticated perturbations.

## 1. Introduction

The most recent global cancer statistics show that lung cancer is the most frequently diagnosed cancer, with 2.5 million new cases (12.4% of all cancers globally), and the leading cause of cancer deaths, with an estimated 1.8 million deaths (18.7% of all cancer deaths) [1]. Given the various treatment options for cancer, radiation therapy remains an important component with approximately 50% or more of all cancer patients receiving radiation therapy during their course of illness [2,3]. For lung cancer, the need for radiotherapy is even higher, up to 77% [4]. One of the challenges facing the radiotherapy community for lung cancer and other cancers of the abdomen and thorax is the management of motion. This has been the subject of several studies [5,6,7,8,9,10]. The novel PET LINAC considered in this paper is among the latest innovations developed to address amongst other issues the motion management problem in radiotherapy.

Some cancers exhibit high metabolic activity, meaning that when injected with a positron emission tomography (PET) radiotracer such as F18-Fluorodeoxyglucose (F18-FDG, or simply FDG), there will be preferential uptake that will clearly make this region appear bright on the PET scan [11]. Recent advances in the field of radiotherapy have resulted in a treatment modality that takes advantage of the biology of such PET-avid tumors to design a treatment that guides radiation beams in real time to irradiate such tumors [12,13,14,15]. This is the novelty of the RefleXion X1 SCINTIX ™ system (RefleXion Medical Inc., Hayward, CA, USA). The system is currently cleared by the U.S. Food and Drug Administration (FDA) to treat PET-avid cancers of the lung and bone using FDG as the radiotracer via the biology-guided radiation therapy (BgRT) concept [16,17,18]. Other sites and radiotracers are actively being investigated for future inclusion [18,19,20,21]. Given that the system itself is a linear accelerator (LINAC), it can also be used to treat any site in the body if the BgRT concept is not used. The technical composition and workflow involved with this system have been described in detail in several publications [12,19,20,21,22,23,24,25,26], but we will present a summary here.

The RefleXion™ X1 SCINTIX™ system is a sophisticated, all-in-one radiotherapy machine that integrates a compact linear accelerator (LINAC) for slice therapy, a kilovoltage computerized tomography (kVCT) unit for fan-beam imaging, and a positron emission tomography (PET) unit for both target localization and radiation delivery guidance. These elements are mounted on a rapidly rotating gantry with unprecedented speeds of 60 RPM, allowing for advanced treatment capabilities.

At the heart of the system is a powerful single-energy LINAC of 6 MV, flattening-filter-free (FFF), that delivers a high-dose-rate photon beam at 1000 cGy/min. This LINAC is equipped with a highly advanced binary multi-leaf collimator (MLC) that can rapidly shape the radiation beam, with leaves that can switch between open and closed states in approximately 7 ms. For patient positioning and alignment, the system includes a 16-slice kVCT scanner that acquires detailed 3D fan-beam images.

A key innovation of the X1 SCINTIX™ system is its integration of PET technology for biology-guided radiotherapy (BgRT). This approach uses PET emissions from the tumor itself to guide the delivery of radiation in real time. The system features two 90-degree PET detector arcs that are integral to the BgRT workflow, which involves using the PET detectors at three critical points: for initial treatment planning, for a pre-scan to ensure consistency before radiation delivery, and during the treatment itself to actively guide the therapeutic beam. The PET scintillators are shielded to reduce interference from scattered radiation. This allows the system to track and treat tumors with exceptional accuracy, even when they are in motion.

The RefleXion X1 SCINTIX ™ system can be used as a regular medical LINAC for image-guided radiotherapy (IGRT) via kVCT only. In this case, the PET subsystem is not utilized at all, and this is also referred to as non-BgRT treatment. This work will focus on the BgRT utility, where currently only lung and bone cancers have been cleared by the FDA. Also, the only radiotracer that has been approved as of now is FDG. In performing treatment planning for a BgRT case, the current system provides two options for the user to categorize target movement, namely periodic and non-periodic motion. In our practice, lung tumors, characterized by respiratory-induced tumor motion, are considered periodic while bone tumors that are rigid and characterized by involuntary motion are classed as non-periodic. This work is focused on lung BgRT.

The ideal candidate for lung BgRT will be one with a periodic and reproducible tumor trajectory from simulation and throughout treatment delivery. The system is designed to support minor deviations from the baseline associated with both tumor motion trajectory and variability in tumor-to-background PET signal changes via a bounded dose volume histogram (bDVH) analysis. The bDVH shows a range of possible curves per region of interest (ROI) rather than a single curve by calculating the best-case- and worst-case-scenario DVH curves, accounting for potential tumor motion (±5 mm) and tumor-to-background PET signal changes (±25%). Another category, where the deviation this time may be due to unwanted patient movement, can lead to an instant perturbation in the baseline breathing pattern. In this case, the trajectory would be characterized by a combination of periodic and non-periodic motion during treatment delivery which was absent and unaccounted for at the time of simulation, FM and pre-PET imaging evaluation sessions. This latter category is the focus of this work.

The unwanted perturbation investigated here is a baseline shift in the tumor’s mean position, induced by a brief, involuntary event like a sneeze, after which the patient’s periodic respiratory motion resumes from this newly displaced baseline. Given the potential for severe dosimetric consequences from a geometric miss in high-dose and high-dose-gradient treatment, understanding system performance when this happens is crucial. To this end, a phantom study was designed to simulate the entire BgRT workflow and evaluate the system’s response to these specific motion perturbations. In particular, a phantom with PET activity on a programmable motion platform was used, where various periodic and non-periodic trajectories were carefully introduced and the robustness of the BgRT system was assessed via the measured dose delivered to the phantom. To the best of our knowledge, this is the first study assessing the impact of perturbation-driven deviations during multi-pass serial tomotherapy BgRT.

## 2. Materials and Methods

### 2.1. Overview of the BgRT Clinical Workflow

An overview of the BgRT workflow from consultation and assessment of initial eligibility up to the final treatment is essential to understanding this work and we present a summary here.

#### 2.1.1. Initial Eligibility at the Time of Patient Consultation

There are five major items to assess at the time of consultation: (a) The tumor must be PET-avid, which can be easily assessed using the diagnostic PET image available at this time. (b) The target standard uptake value (SUV) of 6 is desirable; the higher the better. (c) Tumor size requires a maximum dimension of the order of 2 cm to 5 cm, and ideally between 3 and 4 cm. (d) Patient weight must not exceed 392 lbs, which is a limitation due to the maximum weight the system couch can handle. (e) Finally, an initial qualitative assessment should be done to ensure that the treated target is the predominant area of uptake with minor or no other surrounding uptake areas.

#### 2.1.2. CT Simulation

BgRT patients with non-mobile targets undergo CT simulation wherein a thin-sliced 3DCT image is acquired with a slice thickness of 2 mm or less. For mobile targets, a 4DCT image is acquired which, amongst other data, is used to determine the extent of motion. In our current implementation, only tumors with peak-to-peak motion of 2.5 cm or less are eligible. Other images derived from the 4DCT are used in the treatment planning step and include the maximum intensity projection (MIP) image, the phase-based or amplitude-based binned images, namely 0%, 10%, …, 90% image sets.

#### 2.1.3. Preliminary Treatment Planning

A basic treatment plan which does not involve dose calculation is required for the RefleXion unit to be operated to acquire the FM session PET image needed for the actual planning. For this preliminary plan, the biological tracking zone (BTZ) must be defined, and for the lung case the BTZ is the internal target volume (ITV) plus a margin that accounts for day-to-day variability in breathing pattern. We have, in our practice, used a uniform margin of 10 mm. During PET image acquisition, the gross tumor volume (GTV) as visible on the PET image is in essence the time-weighted average position of the target. In terms of the 4DCT, this will correspond with the GTV as contoured on the image set that is representative of the time-weighted average position. For a phantom with a regular sinusoidal breathing pattern, this would be the 70% image set. This is not always true for a real patient where the breathing pattern is not necessarily regular and consistent. Therefore, in our practice, we have required three GTVs to be outlined, namely GTV-70%, GTV-80%, and GTV-90%, contoured on the 70%, 80%, and 90% image sets respectively. By displaying and evaluating these contours following PET image acquisition, the best match to the observed PET-defined target would represent the actual GTV and thus be used further as the gross tumor volume.

#### 2.1.4. Functional Modeling (Imaging-Only) Session

The basic treatment plan created earlier is uploaded at the treatment delivery console and used to perform the functional modeling (FM) session, previously referred to as the imaging-only session. This is the session that culminates in the acquisition of a PET image that serves three purposes: (a) the image is the basis for final patient eligibility assessment for BgRT; (b) the PET image is used for treatment planning, and (c) the PET image serves as a baseline for subsequent PET evaluation for delivery of all fractions.

The PET radiopharmaceutical tracer currently approved by the FDA for use in BgRT treatment is FDG, with a half-life of 109.7 min. It is administered intravenously, adhering to strict guidelines including a recommended elapsed time from injection to the start of the imaging session. The FDG is typically ordered at least 24 h prior to injection time. The following is a sequence of steps used to complete the task on the day of the FM session.

(a) Daily quality assurance, which includes SPC PET (SPC—system performance check) as well as Laser/kVCT/MV/PET isocenter coincidence. The former is a PET-specific constancy check for energy and time resolution while the latter is a RefleXion X1 system geometric integrity check that ensures the fidelity of the relationship among system subcomponents.
(b) Patient clearance, which includes, amongst other things, confirmation that the patient was NPO 6 h prior to injection, and a blood glucose reading within an acceptable range.
(c) The patient is then asked to void and thereafter is administered 15 mCi of FDG. The exact injection time and injected activity are noted and entered into the treatment console computer.
(d) The patient is requested to wait in the uptake room for at least 30 min post-injection, after which the patient is again asked to void in the hot bathroom. The patient is instructed to flush the toilet at least 3 times after voiding.
(e) Patient setup on the treatment couch begins at around 45 min post-injection to allow time for CT image setup which may include adjustments and re-imaging. The final CT image alignment is reviewed and approved before proceeding to the next step, namely PET image acquisition.
(f) The PET image acquisition begins at 1 h post-injection. After the session, radiation surveys of the entire treatment room and all other areas of interest including the hot bathroom, console area, patient waiting area, trash cans, immobilization devices, and hands and feet of all participating staff are completed and documented.

The FM session results in an acquired PET image which is available for review on the treatment console computer but also available for additional analysis on the TPS. Key eligibility concepts determined by analyzing the PET image from the FM session include the activity concentration (AC), the normalized target signal (NTS), and the maximum activity present within a 2 cm annulus of the BTZ (Max_Activity_BTZ + 2 cm_annulus), compared with the maximum activity within the BTZ (Max_Activity_BTZ). AC represents the amount of radioactive tracer activity per unit volume in the BTZ. It is computed using the mean value of PET voxels above an 80% threshold within the BTZ, ensuring a sufficient signal-to-noise ratio (SNR) against a 3 mm background shell. The ratio of the computed AC to the background noise is the NTS. For the patient to be eligible for BgRT treatment, the following targets must be met:

AC ≥ 5 kBq/mL,(1)

NTS ≥ 2.0 (at planning) and ≥2.7 (at treatment)(2)

Max_Activity_BTZ + 2 cm_annulus ≤ 50% × Max_Activity_BTZ(3)

#### 2.1.5. Treatment Planning and Review

The PET image from the FM session, including the average CT from the 4DCT scan, is used as input to the treatment plan for BgRT. Additional structures are included from the preliminary planning step that may be needed in the clinical treatment plan. In treatment planning for BgRT, the two options available for the user to designate the expected motion are periodic, used for the planning and treatment of targets characterized by respiratory-induced tumor motion, such as in lung cancer, and non-periodic, to designate other motion, such as in bone treatment. Treatments on the RefleXion X1 can be single-pass (1-Pass) or 4-Pass serial tomotherapy. In 1-Pass treatment, the target is translated once in its entirety and treated slice-by-slice until the full target has been treated. For 4-Pass serial tomotherapy, this process is repeated back-and-forth four times. All BgRT treatments are 4-Pass serial tomotherapy, while 1-Pass treatment is used for the IGRT, non-BgRT treatments. The final treatment plan is optimized and evaluated using dose volume histograms (DVHs) that are specific to BgRT called bounded DVHs (or bDVHs). The plan quality is also assessed at this time and approved if deemed satisfactory. All treatment plans must meet established guidelines including a satisfactory score on all quality indices and a gamma analysis for patient-specific QA. We underscore that the patient-specific QA for the BgRT plan is performed on a static phantom in the conventional non-BgRT treatment delivery mode. Our institution’s acceptable gamma passing criteria is consistent with AAPM Task Group 218, namely tolerance and action limits of 95% and 90%, respectively, using a gamma criteria of 3% dose difference and 2 mm distance to agreement (DTA) with a 10% dose threshold.

#### 2.1.6. Pre-Treatment Imaging and Treatment Delivery

The PET tracer injection is already detailed in the discussion of the FM session. The same procedure is followed on the day of treatment. The treatment is delivered following established image-guided radiation therapy (IGRT) protocols that utilize RefleXion’s kVCT imaging for initial patient setup immediately followed by a PET image pre-treatment scan. This initial PET scan is to confirm that the uptake and distribution acquired on the day of treatment are consistent with those used to develop the treatment plan within acceptable limits. This is known as PET evaluation which compares this information to the bounds of the bDVH and returns a pass or fail. If it passes, treatment is allowed to proceed after approval. During the delivery of 4-Pass BgRT, limited-time-sampled (LTS) PET images are acquired during treatment delivery and used to provide tracked dose delivery which can account for positional offsets or tumor motion during treatment. A PET image is displayed at the end of each pass and represents the cumulative image acquired throughout the given pass. At the end of the last pass, a full PET image of the full delivery is displayed and represents the cumulative PET image acquired through all four passes. After the therapy is completed, the information about the delivered radiation is captured in the appropriate treatment records and the patient is discharged. The process is repeated for subsequent fractions until all fractions have been delivered.

### 2.2. Phantom Design Including Trajectory of Tumor Motion with and Without Perturbation

The phantom used for this study was a Sun Nuclear Corporation (SNC) ArcCheck with a special insert designed to hold an injectable PET radiotracer or a PET point source. We used a PET point source as the center of the PET-avid target. The phantom assembly was placed on a CIRS programmable dynamic motion platform (SN 008PLX, CIRS Inc., Norfolk, VA, USA). We designed and uploaded 3 types of motion trajectories on the platform: (1) Periodic motion Cos^4^ with a breathing amplitude of 7.5 mm and frequency of 12 breaths per minute, representative of a normal patient breathing pattern with no perturbation throughout the 4-Pass serial tomotherapy treatment, henceforth referred to as N-Case. (2) Periodic motion Cos^4^ with a known simple single perturbation introduced midway through the 4-Pass serial tomotherapy treatment, henceforth referred to as SP-Case. This is representative of a patient who was otherwise breathing normally but at one point sneezed and as a result the whole body was shifted, but the patient still breathed normally. (3) Periodic motion Cos^4^ with complex multiple perturbations introduced once during each of the four passes of the 4-Pass serial tomotherapy treatment, henceforth referred to as CP-Case. This is representative of a patient who was otherwise breathing normally but, in several instances, sneezed or moved and as a result the whole body was shifted.

### 2.3. BgRT Workflow Process in the Phantom Study

We performed CT simulation where the phantom was set up on the dynamic platform uploaded with the Cos^4^ trajectory in the superior/inferior direction. The peak-to-peak amplitude was set at 15 mm, and the breathing frequency was set at 5 s. The MIP, as well as 70%, 80%, and 90% phase image sets, were sent over to the TPS for preliminary planning.

In the TPS, we contoured the GTV-70%, GTV-80% and GTV-90% from the respective phase images while the ITV was contoured on the MIP image set. The BTZ was generated by adding a 10 mm isotropic margin to the ITV. A preliminary treatment plan was completed followed by an FM session which utilized the N-Case trajectory. By comparing the GTV-70%, GTV-80% and GTV-90% with the acquired PET image from the FM session, it was determined that the GTV-70% was optimal, and therefore we proceeded to define a PTV based on a 5 mm uniform expansion of GTV-70%. The final treatment plan was completed with 10 Gy per fraction, meeting all criteria as one would expect for a stereotactic body radiation treatment (SBRT) plan.

**Figure 1:**
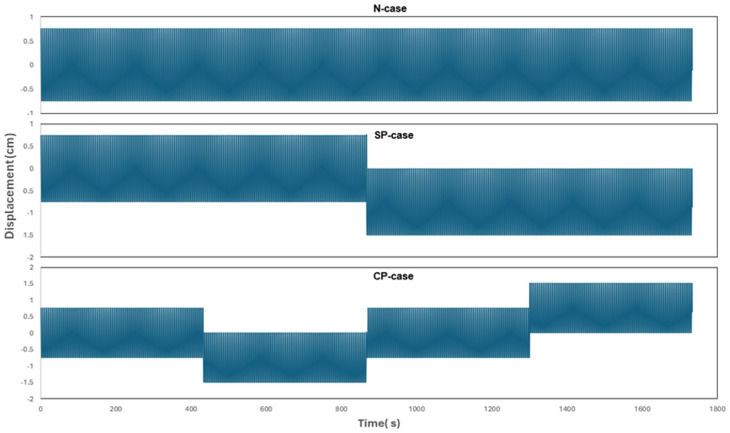
The N-Case (normal breathing pattern) trajectory, the SP-Case—simple (single) perturbation introduced midway through the 4-Pass serial tomotherapy delivery—and the CP-Case—complex (multiple) perturbations where more than one perturbation was introduced, once after each pass of the 4-Pass serial tomotherapy delivery.

We then proceeded to treatment delivery following the workflow as outlined earlier. The treatment delivery workflow was done for three different programmed phantom trajectories, namely the N-Case trajectory representing the baseline as this was the same trajectory used in the FM session, the SP-Case trajectory where a simple perturbation was introduced in an otherwise normal breathing trajectory, and the CP-Case trajectory where a complex trajectory with multiple perturbations was introduced. Each perturbation represented a 7.5 mm displacement in the mean position of baseline motion in the superior–inferior direction (see Figure 1). In each case, the PET evaluation was performed, and the AC, NTS, and dose devliered to the phantom were measured and compared with the planned dose.

It should be noted that, in principle, it is also possible to abort treatment at the end of a given pass when the PET image of that pass is displayed and one determines a misalignment. The next step following this interruption in treatment delivery will be to do a repeat kVCT imaging and correct the misalignment before proceeding to complete the remainder of the treatment. To test the efficacy of this process, we included a fourth category, SP-Case-Corrected, which was the same as the single perturbation case, SP-Case, but with the treatment interrupted immediately following the display of the misaligned PET image in pass 3. Following the interruption, we performed repeat kVCT imaging which allowed us to correct for this perturbation and then continued the treatment delivery, completing the last pass.

### 2.4. Reproducibility and Other Perturbation Studies

To assess how well one could repeat these BgRT experiments and obtain comparable results, we used the SP-Case for illustrative purposes, and on three additional different occasions we set up the phantom and repeated the experiments measuring the dose delivered in each case for comparison. Note that the perturbation studied thus far was a sudden shift of 7.5 mm from the baseline position of an otherwise sinusoidal target trajectory. Using the SP-Case we repeated the delivery with two additional different-magnitude perturbations, namely a 5 mm and a 10 mm shift from the baseline position, and compared the results. All perturbations were chosen such that the target remained within the BTZ for the entirety of the treatment delivery.

## 3. Results

### 3.1. Phantom Design Including Trajectory of Tumor Motion with and Without Perturbation

**Figure 2:**
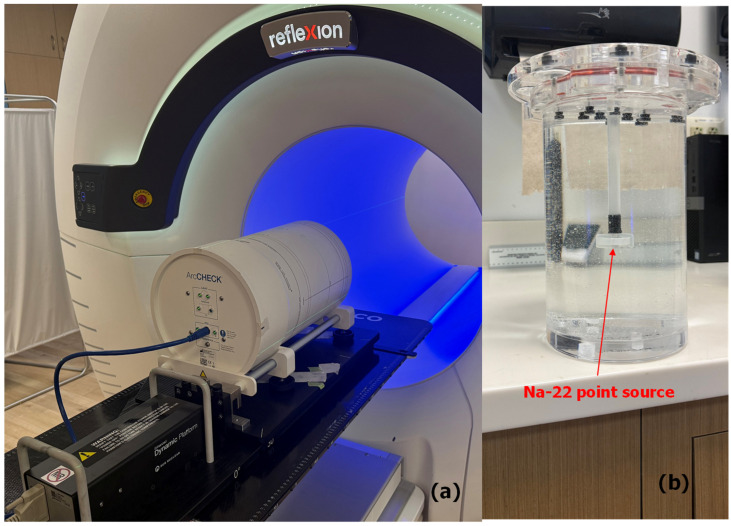
Setup for study: (**a**) SNC ArcCHECK on the CIRS enhanced dynamic platform (SN 008PLX) on the RefleXion X1 couch with (**b**) special insert designed to hold an injectable PET radiotracer. The insert is filled with water.

A picture of the setup is shown in Figure 2, depicting the ArcCheck on the CIRS platform setup on the RefleXion X1 couch. The trajectories used for the phantom studies, N-Case, SP-Case and CP-Case, are as shown in Figure 1.

### 3.2. BgRT Workflow Process with the Phantom Study

**Figure 3:**
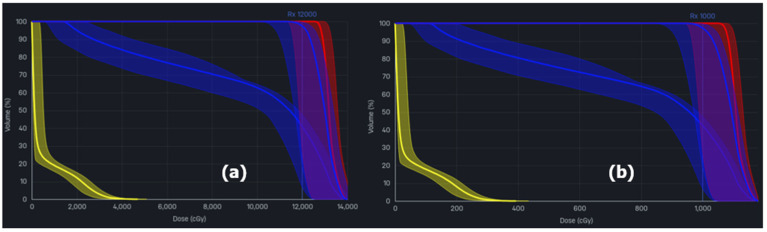
The computed bounded dose volume histogram (bDVH) in (**a**) the plan (10 Gy × 12 fractions) vs. (**b**) the computed bDVH for the N-Case delivery, single-fraction. The bDVHs show a range of possible curves per region of interest (ROI) rather than a single curve by calculating the best-case- and worst-case-scenario DVH curves, accounting for potential tumor motion (±5 mm) and tumor-to-background PET signal changes (±25%).

The eligibility criteria described in Equations (1)–(3) were easily met for our phantom study as a point source was used. Note that this is not always the case for real-world patients. The observed values of AC, NTS and bDVH pass % are summarized in Table 1 below for the three trajectory types, N-Case, SP-Case, and CP-Case, as well as the fourth case that involved an interruption and correction, SP-Case-Corrected. The bDVH for the FM session is comparable with that of the pre-treatment PET image of each of the trajectory cases studied. This is because the perturbations were introduced only during the 4-Pass delivery and, this being a phantom study with point source, nothing was expected to change between the FM session and the day of delivery to affect the bDVH. This is not always the case with a real patient. The bDVH comparison for the FM session versus the N-Case pre-treatment PET imaging is shown in Figure 3 for illustrative purposes.

**Figure 4:**
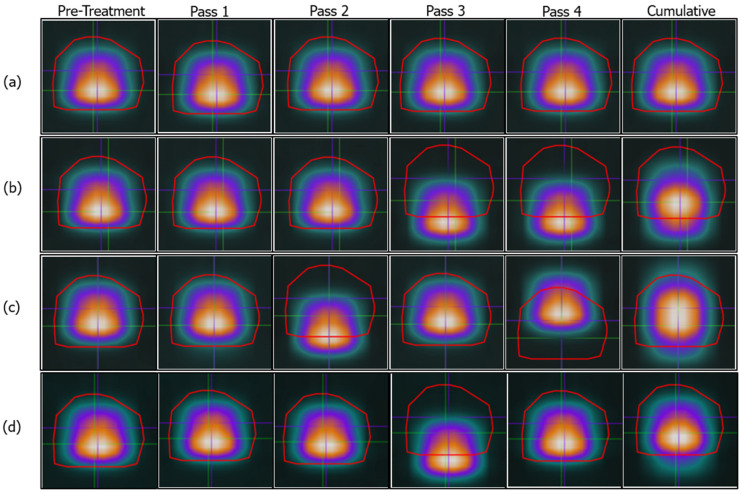
Coronal views of PET images acquired at the start of the treatment (pre-treatment), just before pass 1; the PET images per pass, acquired at the end of each pass; and post-treatment combined PET (cumulative), where the target may have moved based on the introduced known perturbations. The red contours denote the outlines of the GTV for (**a**) N-trajectory, (**b**) SP-trajectory, (**c**) CP-trajectory, and (**d**) SP-Case-Corrected.

As expected for the trajectory cases that involved perturbation, we observed a shifted PET-acquired image when the PET image was acquired after the perturbation event. In Figure 4, we illustrate this by a coronal view of the PET-avid GTV with the GTV contour superimposed on the image. It can be observed in Figure 4 that initially in the workflow, when there is no perturbation introduced to the breathing motion, the acquired PET image aligns with the GTV as expected in the stated areas in N-Case (all of row (a)), SP-Case (pre-treatment, pass 1 and pass 2), CP-Case (pre-treatment, pass1 and pass3), and SP-Case-Corrected (pre-treatment, pass 1, pass 2 and pass 4). As perturbation was introduced in the trajectory and other PET images were acquired after this event, we can observe the displayed image misalignment with the GTV contour (Figure 4, passes 3 and 4 for row (b), passes 2 and 4 for row (c), and pass 3 of row (d)). Note that per the trajectory in Figure 1, the CP-Case self-corrected and returned to baseline in pass 3 which is observed in Figure 4, pass 3 of row (c). The GTV is shown in a fixed frame of reference, and this is why the misalignment is visible. Figure 4 is not an illustration of the dose delivered but rather must be interpreted carefully.

**Figure 5:**
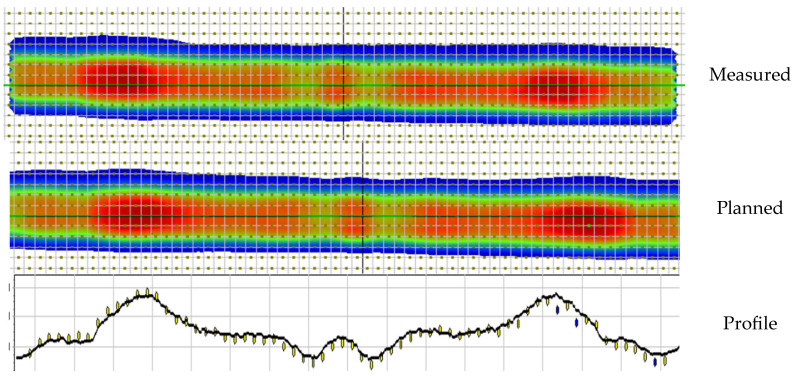
An example pf the measured vs. planned dose used in the gamma analysis on the SNC patient software.

In each of the experiments, N-Case, SP-Case, CP-Case, and SP-Case-Corrected, the delivered dose to the phantom was measured and compared to the planned dose based on there being no perturbations. Using gamma index analysis [27] with a dose threshold of 10% and a dose difference and distance to agreement (DTA) criterion of 3%/3 mm [28], the percentages of points with a gamma less than or equal to 1 and therefore considered as passing were 97.8%, 95.4%, and 98.2% for N-Case, SP-Case, and CP-Case, respectively. The analysis was repeated using other criteria like 3%/2 mm [29], as well as 5%/1 mm [30] and 1%/1 mm, as summarized in Table 2. These would all be considered passing in clinical practice. Note that in comparison and as a reference, results of the routine patient-specific QA performed on a static phantom in the conventional non-BgRT treatment delivery mode show gamma passing rates of 98.8%, 98.8%, 99.6%, and 91.5% using the 3%/3 mm, 3%/2 mm, 5%/1 mm, and 1%/1 mm criteria respectively. We illustrate one of the gamma analysis examples showing the dose comparison and a profile in Figure 5.

We also observed, as shown in Table 2, that the special case, SP-Case-Corrected, where the treatment was interrupted following an observed misaligned PET image after pass 3 delivery and the perturbation was corrected via repeat kVCT imaging before completing the remainder of the treatment delivery, consistently resulted in lower gamma passing rates compared to the uninterrupted counterpart, SP-Case. The gamma passing rates for SP-Case-Corrected were 91.1%, 93.8%, 89.6%, and 48.9% compared with 92.2%, 95.4%, 93.8%, and 56.3% for the SP-Case based on 3%/2 mm, 3%/3 mm, 5%/1 mm, and 1%/1 mm respectively.

### 3.3. Reproducibility and Other Perturbation Studies

We observed excellent reproducibility when we repeated the treatment delivery using the SP-Case as an example. A total of four repeated deliveries were performed, and the measured dose was very comparable in each case. The average gamma passing rates considering all repeat measurements were 97.3% ± 1.6% (range, 95.4% to 99.4%), 95.2% ± 2.2% (range, 92.2% to 98.2%), 96.9% ± 1.8% (range, 93.8% to 98.4%), and 70.2% ± 8.4% (range 56.3% to 79.0%) based on the criteria 3%/3 mm, 3%/2 mm, 5%/1 mm, and 1%/1 mm respectively.

When we introduced a lower (5 mm) and a higher (10 mm) perturbation compared to the 7.5 mm perturbation that had so far been studied, we observed, using the SP-Case as an example, that the measured gamma passing rates were very comparable, indicating that the system would handle different perturbations in the same manner. The measured differences in gamma passing rate when the 5 mm perturbation was introduced compared with the 7.5 mm perturbation for the SP-Case were 0%, −0.6%, 2.4%, and 14.1% based on the criteria 3%/3 mm, 3%/2 mm, 5%/1 mm, and 1%/1 mm respectively. In a similar analysis, the measured differences in gamma passing rate when the 10 mm perturbation was introduced compared with the 7.5 mm perturbation for the SP-Case were 0.2%, −1.3%, 1.4%, and 17.3% based on the criteria 3%/3 mm, 3%/2 mm, 5%/1 mm, and 1%/1 mm respectively.

## 4. Discussion

The RefleXion X1 SCINTIX ™ system is a novel PET LINAC with BgRT capability in the periodic and non-periodic modes. The expected motion associated with the target trajectory informs the user as to what mode is selected for use in the entire workflow starting from the treatment planning step. Lung BgRT, characterized by respiratory-induced target motion, would be planned as periodic, whereas the BgRT of rigid body targets such as bone would be non-periodic. For lung cancer BgRT, the target trajectory, even for ideal cases that start off regular and periodic, may occasionally experience unwanted perturbations that were unplanned and unaccounted for during the treatment planning step. Examples in real life that may introduce such perturbations may include coughing, sneezing, arm, leg, and/or whole-body movement, etc. The goal of our work was to assess whether, given such perturbations, the system would adapt and still deliver the correct dose to the patient.

To do this, phantom experiments were designed to go through the elaborate BgRT workflow, delivering a 4-Pass serial tomotherapy BgRT treatment and measuring the dose in three different scenarios: (1) the N-Case trajectory, characterized by regular sinusoidal target motion with no perturbation introduced in the phantom trajectory, (2) the SP-Case where the same treatment plan was delivered, but with a single perturbation introduced midway through the delivery, right after the second pass of the 4-Pass plan, and finally the (3) CP-Case where four complex perturbations were introduced in the course of delivery, one at each pass of the 4-Pass serial tomotherapy delivery. We limited the perturbation study to 1D, in the superior/inferior direction, for simplicity. A 3D study would be more involved and include the design of an advanced programmable phantom where perturbations in the anterior/posterior and left/right directions are automated as well. This would be the subject of future work.

The eligibility for BgRT treatment was assessed at the beginning of each phantom experiment, including calculation of AC, NTS, and bDVH % pass rate. Given that a point source was used with no other activity in nearby areas, eligibility based on these criteria index calculations was always easily met. By measuring the total dose delivered in the course of each phantom experiment and comparing it to the expected dose without perturbation, the robustness of the system to adapt and still deliver an accurate dose to the target in such circumstances was readily assessed. Adopting the gamma analysis for dose comparison between the planned and measured doses, we observed gamma passing rates of 97.8%, 95.4% and 98.2% for the N-Case, SP-Case, and CP-Case respectively. These results, although lower for the SP-Case, would all be acceptable clinically, and therefore with respect to the specific perturbation studied in this work, namely a sudden shift of up to 10 mm from the baseline position of an otherwise sinusoidal target trajectory, one can state that the system is robust and adaptable, able to deliver the dose accurately. We emphasize that although acceptable in this perturbation study, the point source design is not representative of the uptake in a real patient. To design such a phantom, one must consider, at minimum, a phantom design that accounts for uptake from both the target and the background surrounding areas. We utilized the same phantom as part of the BgRT end-to-end commissioning experiment where FDG was injected into a phantom target and a diluted form into the surrounding for background emissions. In such advanced phantom studies, the calculated AC, NTS, and bDVH would be very close to the threshold values as opposed to the very high numbers reported here based on the point source design.

**Figure 6:**
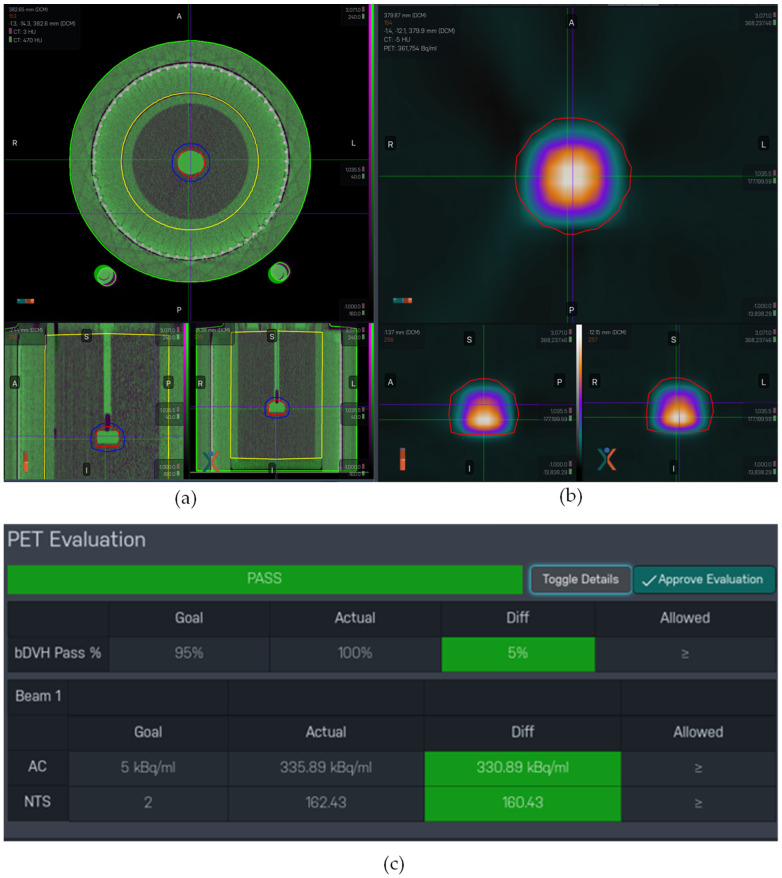
Illustration of setup for the N-Case normal breathing trajectory where (**a**) in the initial step of the workflow, the acquired kVCT image is aligned with the reference CT image and shifts are determined to re-align the patient. In (**b**), the PET acquisition is the next step, where the PET image is acquired, and as seen with the displayed GTV-70% image in red, this aligns well with the target. The PET image is then used in the PET evaluation which computes a bounded DVH with a bDVH pass %, and the AC and NTS are also computed. (**a**) Alignment of acquired kVCT vs. reference; (**b**) acquired PET image; (**c**) PET evaluation showing bDVH pass rate, AC and NTS.

The relatively lower gamma passing rate for the SP-Case compared with the N-Case or the CP-Case, which becomes more evident as the gamma criteria are tightened (3%/2 mm, 5%/1 mm, 1%/1 mm), can be explained by taking a closer look at Figure 1, Figure 3 and Figure 6 and how these are introduced. For the single simple perturbation, SP-Case, a one-sided and unrecoverable perturbation was introduced midway through the 4-Pass BgRT, whereas for the CP-case, although it looked complex, the perturbation introduced earlier at the end of the first pass was immediately recoverable at the end of the second pass and the final perturbation was in the opposite direction as the first. One would therefore expect a worse gamma passing rate for the SP-Case, which is what was observed and became more evident as we tightened the gamma criteria.

The special case, SP-Case-Corrected, in which we interrupted treatment to correct an observed misalignment based on the PET image displayed at the end of a pass, is of practical clinical interest and it is important to explore this further. There are two main issues with this approach. First, a PET image is only displayed at the end of each pass; therefore, if the perturbation occurred at time (To) for a pass with duration (*T*), then by virtue of this process, a portion of the treatment (*T*-*To*) would have been delivered without the intended correction. Second, given that the displayed PET image is cumulative, for the perturbation to be visible on the pass image, To must be small compared with *T*. Nevertheless, although more time-consuming and inefficient in the patient delivery process, the expectation was that this approach would be better or at least comparable with the SP-Case where the system corrected for the perturbation via the BgRT method of dose tracking. It turns out that this was not the case. The gamma passing rate for SP-Case-Corrected was consistently lower than the gamma passing rate for the SP-Case where the system was uninterrupted. The observed reductions were 1.1%, 1.6%, 4.2%, and 7.4% based on a 10% dose threshold and gamma criteria of 3%/2 mm, 3%/3 mm, 5%/1 mm, and 1%/1 mm respectively. Although more studies are needed before any definite conclusions can be made, this single study suggests that it may be better to leave the system uninterrupted when the perturbations occur. It is important that clinical teams perform their own internal studies to support their chosen approach.

We used a gamma criterion of 3%/3 mm and a 10% dose threshold as a basis for the clinical acceptability of these scenarios. We underscore that we would still deem all the cases acceptable if we used the tighter criterion 3%/2 mm based on the American Association of Physicists in Medicine Task Group number 218 (AAPM TG-218) [29]. The AAPM Task Group report is based on patient-specific QA on a static phantom and considers 95% as a tolerance limit for passing with 90% as the action limit. The phantom deliveries in this work were more involved as our study included dose delivery on a moving phantom. Even then, the gamma passing rate in each case was above the action limit.

Finally, we summarize some limitations and future directions related to this work. The current study was a phantom investigation involving perturbation in 1D only. To closely mimic an actual patient, a future direction would be to consider a sophisticated phantom capable of automating any desired perturbation in 3D. We also used a point source which simplified our setup and always met the AC, NTS, and bDVH eligibility criteria. A future direction would be to consider a phantom with FDG injected into both the target and surrounding medium to create an uptake and PET emission distribution that is closer to a real patient. We used a smooth sinusoidal curve for periodic motion in 1D. A future consideration would be to incorporate an actual trajectory from a real patient that is not necessarily smooth with constant amplitude and frequency over the entirety of the treatment. In all the experiments, we used ArcCheck on a moving platform for dose measurements. Radiochromic film with a superior spatial resolution could be considered for future studies where a tighter gamma evaluation criterion like 1%/1 mm, especially for plans with high dose gradients, is desired. Other considerations such as target deformation and/or latency effects are equally important but may be more challenging to incorporate in a phantom study.

## 5. Conclusions

A phantom investigation of the robustness and adaptability of the novel RefleXion X1 SCINTIX^TM^ system to perturbations introduced by unplanned non-periodic shifts in an otherwise periodic target trajectory when delivering a 4-Pass BgRT was presented. By comparing the dose delivered under a normal periodic sinusoidal tumor motion to the dose delivered under perturbed sinusoidal delivery, one with a simple perturbation introduced at the end of the second pass of a 4-Pass BgRT delivery and another more complex situation with perturbation introduced at each of the four passes of a 4-Pass BgRT delivery, we observed acceptable gamma passing rates, greater than 95% in each scenario based on a 10% dose threshold and a 3%/3 mm criterion, thus suggesting that the system is robust and adaptable, able to deliver the correct dose under such perturbations. This was based on a specific programmed perturbation of an up to 10 mm shift in the average target position from baseline in the superior/inferior direction of an otherwise periodic trajectory. More work needs to be done to include additional scenarios of perturbation before a general conclusion can be drawn.

## Figures and Tables

**Table 1:** Summary of the activity concentration (AC), normalized target signal (NTS) and bounded dose volume histogram (bDVH) pass % for the experimental deliveries.

Trajectory Type	AC	NTS	bDVH Pass %
N-Case ^1^	335.89 kBq/mL	162.43	100%
SP-Case ^2^	318.16 kBq/mL	182.71	100%
CP-Case ^3^	320.46 kBq/mL	205.35	100%
SP-Case-Corrected ^4^	304.13 kBq/mL	185.83	100%

^1^ Normal breathing pattern. ^2^ Single perturbation introduced midway through the 4-Pass serial tomotherapy. ^3^ Multiple complex perturbations introduced after passes 1, 2, and 3. ^4^ Single perturbation introduced midway through the 4-Pass serial tomotherapy; system aborted when observed; repeat imaging performed and corrected for the remainder of the treatment.

**Table 2:** Summary of results of gamma analysis using ArcCHECK.

Trajectory Type	Gamma (3%/2 mm)	Gamma (3%/3 mm)	Gamma (5%/1 mm)	Gamma (1%/1 mm)
N-Case	96.6%	97.8%	98.4	57.8%
SP-Case	92.2%	95.4%	93.8%	56.3%
CP-Case	97.0%	98.2%	98.4%	71.5%
SP-Case-Corrected	91.1%	93.8%	89.6%	48.9%

## Data Availability

The raw data supporting the conclusions of this article will be made available by the authors on request.
